# Transmission Characteristics Analysis and Compensation Control of Double Tendon-sheath Driven Manipulator

**DOI:** 10.3390/s20051301

**Published:** 2020-02-27

**Authors:** Haoting Wu, Meng Yin, Zhigang Xu, Zhiliang Zhao, Wei Han

**Affiliations:** 1 Shenyang Institute of Automation, Chinese Academy of Sciences, Shenyang 110016, China; zgxu@sia.cn (Z.X.); 1870267@stu.neu.edu.cn (Z.Z.); hanweiZYB@163.com (W.H.); 2 Institutes for Robotics and Intelligent Manufacturing, Chinese Academy of Sciences, Shenyang 110169, China; 3 University of Chinese Academy of Sciences, Beijing 100049, China; 4 School of Mechanical Engineering and Automation, Northeastern University, Shenyang 110169, China

**Keywords:** tendon-sheath drive, robot, compensation control, trajectory tracking

## Abstract

The double tendon-sheath drive system is widely used in the design of surgical robots and search and rescue robots because of its simplicity, dexterity, and long-distance transmission. We are attempting to apply it to manipulators, wherenon-linear characteristics such as gaps, hysteresis, etc., due to friction between the contact surfaces of the tendon sheath and the flexibility of the rope, are the main difficulties in controlling such manipulators. Most of the existing compensation control methods applicable to double tendon-sheath actuators are offline compensation methods that do not require output feedback, but when the system’s motion and configuration changes, it cannot adapt to the drastic changes in the transmission characteristics. Depending on the transmission system, the robotic arm, changes at any time during the working process, and the force sensors and torque sensors that cannot be applied to the joints of the robot, so a real-time position compensation control method based on flexible cable deformation is proposed. A double tendon-sheath transmission model is established, a double tendon-sheath torque transmission model under any load condition is derived, and a semi-physical simulation experimental platform composed of a motor, a double tendon-sheath transmission system and a single articulated arm is established to verify the transfer model. Through the signal feedback of the end encoder, a real-time closed-loop feedback system was established, thus that the system can still achieve the output to follow the desired torque trajectory under the external interference.

## 1. Introduction

A tendon-sheath is a flexible transmission mechanism composed of an internal flexible cable and an external hollow casing. Due to the friction between the sleeve and the flexible cable, and as well as elastic deformation of the flexible cable after tension, the transmission system will have gaps, hysteresis, and dead zones during the working process, which will affect the transmission effect. This is especially noticeable when the direction needs to be changed frequently, as it is difficult to improve the control accuracy of the system. Therefore, in the application process, it is necessary to reduce the influence of the friction link in the tendon-sheath drive. The double tendon-sheath transmission system uses a pull-pull structure for power transmission, which solves the problem of unidirectionality in the single tendon-sheath transmission system. However, this still it involves the coupling of two tendon-sheaths during the transmission process, which makes the analysis of the transmission system more complex.

Regarding research on tendon-sheath driving systems, feedforward control is usually used in the application of surgical medical equipment to improve the tracking accuracy. For manipulators with larger structural sizes, feedforward control is not suitable for solutions requiring higher-cost torque sensors on the execution side and the installation of tension sensors on the motor side. Therefore, the encoder is fixed to the joint, and a reasonable and simple control model needs to be selected on the basis of ensuring reducing costs.

The hysteresis of the tendon sheath mechanism is very complex, and many models are limited to specific situations. Kaneko et al. [1] determined the behaviour of hysteresis and the clearance of tendon-sheath transmission system, established a static model of a single tendon-sheath transmission and a dynamic model of lumped parameters using a Coulomb friction model, and introduced a control strategy to eliminate directional dependence. Phee et al. [2] designed a dual-arm master-slave robotic surgery system for oral endoscopic surgery, where each joint is driven by two separate tendon-sheathes. The displacement sensor and the force sensor at the input end is used to achieve the end output force and an estimate of the location. Nguyen et al. [3] developed a mechanical foot for footwear testing using a tendon-sheath drive mechanism and adopted an offline compensation control method to improve the system performance without an output sensor. Tian and Wang [4], Phee et al. [5], Chen et al. [6], and Wu et al. [7] used the Coulomb friction model to characterize the force/motion transmission of the tendon-sheath drive system. Subsequently, the inverse drive model and the control were used to compensate for the friction, but these methods usually assume that the casing is fixed, which is difficult to achieve in such practical applications as robotic arms. The adaptive algorithm is more suitable for the hysteretic modelling of tendon-sheath mechanisms (TSMs). Li et al. [8,9] proposed a deep learning method that predicts the far-end force of TSMs based on the near-end measurements and can achieve accurate predictions in experiments with constant system speeds. However, these methods usually assume that the casing is fixed, which is difficult to achieve in the application of actual robotic arms. Agrawal et al. [10] utilized partial derivative equations to describe the transmission characteristics of the tendon-sheath drive without the assumption of a constant preload or a fixed casing. Its complex model parameters and discontinuities increased the computational burden, thereby limiting the use of this method. Another method for modelling the transmission characteristics of a tendon-sheath transmission is to use the inverse model [11,12] or the similar inverse lag model [13,14] to obtain the transfer curve, and then use feedforward control to compensate for the friction and clearance. Although the offline compensation method does not require output feedback and is suitable for systems where sensors cannot be installed remotely, the system cannot cope with the drastic changes in transmission characteristics when the system’s pretension and tendon-sheath configuration change during operation, thereby limiting the tracking control performance.

Using feedback outputs such as image processing systems, researchers can use closed-loop control algorithms to improve the position tracking performance. Do et al. [15,16,17] used the Bouc-Wen model [18] to describe the motion transmission with the displacement input, and developed an adaptive control scheme to respond to changes in the tendon-sheath configuration and perform position tracking control, however, no torque control algorithm was proposed. Kong et al. [19] developed a tendon sheath-driven serial elastic actuator (RSEA) that operates under the torque mode control. The system only uses position feedback and a flexible cable tension to compensate for the frictional force of the tendon-sheath bending. Lu et al. [20] obtained the offline estimation of the inertia and the damping of the geared motor from the deformation of the spring and derived output torque from the disturbance observer (DOB). The tendon sheath-driven RSEA achieved output torque control through artificially assisted control joints. Zhang et al. [21] performed torque mode control research on 9 low-level controllers and 3 high-level controllers to determine the best control scheme for driving the ankle exoskeleton in assisting people’s ankle walking. However, the system uses the feedback extension of the elastic element (that is, the series spring) to derive the output torque, and uses a model-free control law to track the torque curve, regardless of the nonlinear transmission characteristics of the tendon-sheath drive system.

Schiele et al. [22] studied the characteristics of double tendon-sheath transmission and applied it to a wearable exoskeletons to study the transmission characteristics between robot joints and a actuators. The system has a lower resistance for movement during spontaneous movement and higher torque during hard contact. Agrawal et al. [23,24] proposed an analytic model of a double tendon-sheath transmission system, which is characterized by a set of partial differential equations (PDE) in the continuous-time domain. In theory, it can be used to analyse systems with gaps, double tendon-sheath coupling, prestressing, and curvature. However, the derivation model represented by a set of PDEs is deemed to be based on a specific environment, thus it is not suitable for the real-time control of actual robots.

Lai et al. [25,26] studied the haptic feedback system of a flexible endoscopic surgical robot and proposed a single-axis fibre Bragg grating (FBG)-based force sensor for a TSM of a robotic arm, as well as an integrated sensor-model approach to estimate the forces on other TSMs of that arm.The sensor-model approach could accurately estimate the distal force with an RMSE of 0.65 N. The proposed approach and sensor can also be applied for a variety of TSMs-driven systems, such as robotic fingers/hands, wearable devices, and rehabilitation devices.

When a tendon-sheath actuator (TSA) is applied to a robotic arm, it has a large structural size and a wide range of motion, and it is difficult to install the force sensor on the motor side. With an increase in the robot’s task requirements, especially the contact performance of human-computer interaction, which have higher requirements, and during the movement of the humanoid manipulator driven by the double tendon-sheath, the configuration of the tendon-sheath transmission system can change at any time. Therefore, the reliability of detecting the signal at the end in the real-time is greater than the off-line compensation control method. Taking all of these factors into consideration, a systematic method of a single TSA torque estimation based on a position transfer model is proposed. A semi-physical simulation experimental platform for driving a single articulated arm with a double tendon-sheath transmission system is established. The correctness of the transfer model is then verified through experiments, and the closed-loop feedback position control of the joint rotation angle is obtained through real-time feedback of the end position signal. When the external configuration environment changes, the output torque can still follow the desired torque trajectory.

## 2. Analysis of the Double Tendon-sheath Transmission Characteristics

**Figure 1:**
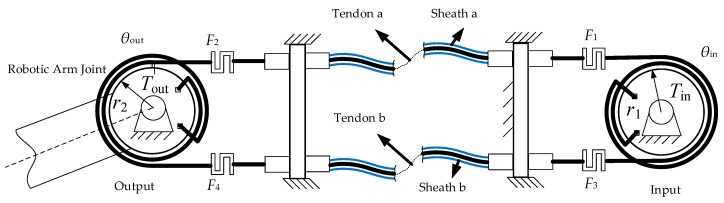
Model of the double tendon-sheath drive system.

In the humanoid robotic arm, a double tendon-sheath transmission is used to drive the robot arm joints for both movement and control. In this system, after the flexible cable passes through the sleeve, the two sides are, respectively, fixed on the pulleys at the input end, and the output end and the servo motor transmits the torque to the output end through the tendon-sheath transmission. There is friction between the tendon and the sheath, and the tendon is deformed by the tensile force. Therefore, the tendon-sheath driving system is modeled from these two aspects. The double tendon-sheath transmission system model is shown in Figure 1.

Since the radius of the pulley is much smaller than the length of the tendon-sheath, during the movement of the robotic arm, it can be assumed that the curvature changes of the two tendon-sheaths along the length in the same double tendon-sheath system are the same. At a certain time t, the full curvature *Κ* of the tendon-sheath is defined as

(1) $$Κ\left(t\right)=\int_{0}^{L}\kappa \left(\sigma ,t\right)d\sigma$$

where *κ*(*σ*,*t*) is the curvature of the tendon-sheath. Assuming that the average curvature of the tendon-sheath at the corresponding time is *κ*, the full curvature at this time can be expressed as *Κ* = *κL*. When $\dot{\upsilon }\left(l,t\right)$ is 0, the output tension and the elongation of the double tendon-sheath transmission system are the same as in the previous moment. Therefore, when analysing the characteristics of the double tendon-sheath transmission, only the process of non-zero motion speed needs to be derived.

When the robotic arm rotates clockwise, the force transmission relationship between flexible cable a and flexible cable b is

(2) $$F_{2}\left(t\right)=F_{1}\left(t\right)\exp\left(-\mu \Theta \right)=F_{1}\left(t\right)Q_{p}$$

(3) $$F_{4}\left(t\right)=F_{3}\left(t\right)\exp\left(\mu \Theta \right)=F_{3}\left(t\right)Q_{r}$$

where *Q*_p_ = exp(−*μΚ*), and *Q*_r_ = exp(*μΚ*).

The input torque and the output torque in a double tendon-sheath transmission system can be expressed as the product of the radius of its corresponding side pulley and the difference in the pull-up force of the flexible cable.

(4) $$T_{\mathrm{in}}\left(t\right)=\left[F_{1}\left(t\right)-F_{3}\left(t\right)\right]r_{1}$$

(5) $$T_{\mathrm{out}}\left(t\right)=\left[F_{2}\left(t\right)-F_{4}\left(t\right)\right]r_{2}$$

The elongations of cords a and b are

(6) $$\Delta L_{a}\left(t\right)=r_{1}\theta_{\mathrm{in}}\left(t\right)-r_{2}\theta_{\mathrm{out}}\left(t\right)$$

(7) $$\Delta L_{b}\left(t\right)=r_{2}\theta_{\mathrm{out}}\left(t\right)-r_{1}\theta_{\mathrm{in}}\left(t\right)$$

Considering the elastic modulus and the cross-sectional area of the flexible cable, the elongation of a flexible cable a and flexible cable b can be expressed as

(8) $$\Delta L_{a}\left(t\right)=\frac{F_{1}\left(t\right)\psi_{p}-F_{0}L}{EA}$$

(9) $$\Delta L_{b}\left(t\right)=\frac{F_{3}\left(t\right)\psi_{r}-F_{0}L}{EA}$$

where *Ψ*_p_ = (*L*/*μΚ*)(1 − *Q*_p_); *Ψ*_r_ = (*L*/*μΚ*)(1 − *Q*_r_).

It can be determined from Equations (8) and (9) that.

(10) $$F_{1}\left(t\right)=\left[F_{3}\left(t\right)\psi_{r}+2F_{0}L\right]/\psi_{p}$$

From Equations (4) and (10), the relationship between the cable tension at the output and the motor input torque is

(11) $$F_{1}\left(t\right)=\frac{\psi_{r}T_{\mathrm{in}}\left(t\right)-2F_{0}Lr_{1}}{r_{1}\left(\psi_{r}-\psi_{p}\right)}$$

(12) $$F_{3}\left(t\right)=\frac{\psi_{p}T_{\mathrm{in}}\left(t\right)-2F_{0}Lr_{1}}{r_{1}\left(\psi_{r}-\psi_{p}\right)}$$

The relationship between the tension of the flexible cable on the remotely driven wheel and the input torque of the motor is

(13) $$F_{2}\left(t\right)=\frac{\left[\psi_{r}T_{\mathrm{in}}\left(t\right)-2F_{0}Lr_{1}\right]}{r_{1}\left(\psi_{r}-\psi_{p}\right)}\left(1-\psi_{p}\frac{Κ}{L}\right)$$

(14) $$F_{4}\left(t\right)=\frac{\left[\psi_{p}T_{\mathrm{in}}\left(t\right)-2F_{0}Lr_{1}\right]}{r_{1}\left(\psi_{r}-\psi_{p}\right)}\left(1-\psi_{r}\frac{Κ}{L}\right)$$

Therefore, the relationship between the remote output torque and the remote motor input torque is

(15) $$T_{\mathrm{out}}\left(t\right)=\frac{r_{2}T_{\mathrm{in}}\left(t\right)}{r_{1}}-2F_{0}r_{2}\mu Κ$$

When the direction of movement is reversed

(16) $$T_{\mathrm{out}}\left(t\right)=\frac{r_{2}T_{\mathrm{in}}\left(t\right)}{r_{1}}+2F_{0}r_{2}\mu Κ$$

When the rotation speed of the remote pulley is 0, the torque transmission relationship is

(17) $$T_{\mathrm{out}}\left(t\right)=\left\{ \begin{array}{l} \frac{r_{2}T_{\mathrm{in}}\left(t\right)}{r_{1}}-\mathrm{sign}\left(\omega_{\mathrm{in}}\right)2F_{0}r_{2}\mu Κ,\omega_{\mathrm{in}}\left(t\right)\neq 0\\ T_{\mathrm{out}}\left(t^{-}\right),\omega_{\mathrm{in}}\left(t\right)=0 \end{array}\right.$$

It can be determined from the above analysis that there are many factors that affect the transmission characteristics during the movement of the double tendon-sheath transmission system. The main influencing factors are the pretension force, the full curvature of the tendon-sheath, the friction factor between the sleeve and the flexible cable, the pulley’s radius, the transmission direction, etc. It can be seen from Equation (17) that the maximum resistance torque of the casing to the flexible cable is –sign(*ω*_in_)2*F*_0_*r*_2_*μΚ*. If ∣*T*_in_*r*_2_/*r*_1_ − *T*_out_ ≤ 2*F*_0_*r*_2_*μΚ*∣, then the system is at a standstill and the output torque is the same as the previous moment; if ∣*T*_in_*r*_2_/*r*_1_ − *T*_out_ > 2*F*_0_*r*_2_*μΚ*∣, then the system enters the transmission state.

According to Equation (15), the relationship between the output angle and the input angle of the double tendon-sheath transmission system can be obtained as

(18) $$\theta_{\mathrm{out}}\left(t\right)=\frac{r_{1}\theta_{\mathrm{in}}\left(t\right)}{r_{2}}-\frac{T_{\mathrm{in}}\left(t\right)\psi_{r}\psi_{p}-F_{0}Lr_{1}\left(\psi_{r}+\psi_{p}\right)}{AEr_{1}r_{2}\left(\psi_{r}-\psi_{p}\right)}$$

When the direction of movement is reversed

(19) $$\theta_{\mathrm{out}}\left(t\right)=\frac{r_{1}\theta_{\mathrm{in}}\left(t\right)}{r_{2}}-\frac{T_{\mathrm{in}}\left(t\right)\psi_{r}\psi_{p}+F_{0}Lr_{1}\left(\psi_{r}+\psi_{p}\right)}{AEr_{1}r_{2}\left(\psi_{r}-\psi_{p}\right)}$$

From Equations (18) and (19), it can be determined that when the direction of movement of the two pulleys is changed, the direction of the force on the flexible cable changes. The movement of the proximal pulley cannot be immediately transmitted to the distal driven pulley.

(20) $$\varphi \left(t\right)=\left\{ \begin{array}{l} \varphi_{s}\left(t\right)=\frac{T_{\mathrm{in}}\left(t\right)\Psi_{r}\Psi_{p}-F_{0}Lr_{1}\left(\Psi_{r}+\Psi_{p}\right)}{AEr_{1}r_{2}\left(\Psi_{r}-\Psi_{p}\right)},\omega_{\mathrm{in}}\left(t\right)>0\\ \varphi_{n}\left(t\right)=\frac{T_{\mathrm{in}}\left(t\right)\Phi_{r}\Phi_{p}+F_{0}Lr_{1}\left(\Psi_{r}+\Psi_{p}\right)}{AEr_{1}r_{2}\left(\Psi_{r}-\Psi_{p}\right)},\omega_{\mathrm{in}}\left(t\right)<0 \end{array}\right.$$

Let *t*_0_ be the last moment when the system turns, and △*θ*_in_ represents the lag angle on the input side.

(21) $$\theta_{\mathrm{out}}\left(t_{0}\right)=\frac{r_{1}}{r_{2}}\theta_{\mathrm{in}}\left(t_{0}\right)-\varphi \left(t_{0}\right)$$

(22) $$\theta_{\mathrm{out}}\left(t\right)=\frac{r_{1}}{r_{2}}\theta_{\mathrm{in}}\left(t\right)-\varphi \left(t\right)$$

(23) $$\Delta \theta_{\mathrm{in}}\left(t\right)=\left\{ \begin{array}{l} \varphi_{s}\left(t\right)-\varphi_{n}\left(t_{0}\right),\omega_{\mathrm{in}}\left(t\right)>0\\ \varphi_{n}\left(t\right)-\varphi_{s}\left(t_{0}\right),\omega_{\mathrm{in}}\left(t\right)<0 \end{array}\right.$$

When △*θ*_in_ is negative, the hysteresis angle is compensated, and we obtain

(24) $$\theta_{\mathrm{out}}\left(t\right)=\left\{ \begin{array}{l} \frac{r_{1}}{r_{2}}\theta_{\mathrm{in}}\left(t\right)-\varphi_{s}\left(t\right),\omega_{\mathrm{in}}\left(t\right)>0\;\mathrm{and}\;\Delta \theta_{\mathrm{in}}\left(t\right)<0\\ \frac{r_{1}}{r_{2}}\theta_{\mathrm{in}}\left(t\right)-\varphi_{n}\left(t\right),\omega_{\mathrm{in}}\left(t\right)<0\;\mathrm{and}\;\Delta \theta_{\mathrm{in}}\left(t\right)<0\\ \theta_{\mathrm{out}}\left(t^{-1}\right),\mathrm{else} \end{array}\right.$$

## 3. Model Validation

### 3.1. Experimental Device for a Double Tendon-Sheath Transmission System

**Figure 2:**
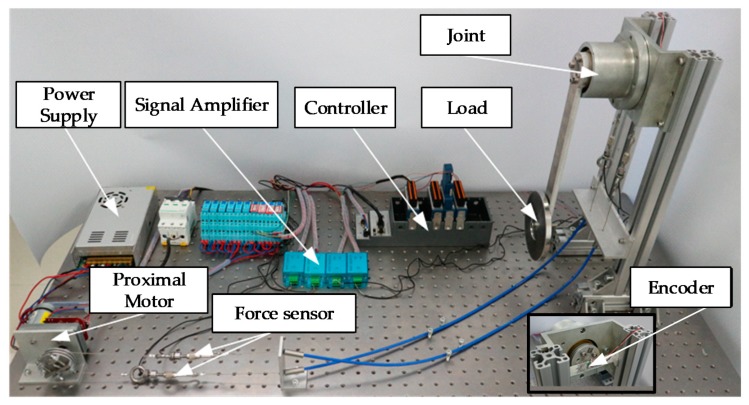
Double tendon-sheath drive test bench.

To verify the correctness of the double tendon-sheath transmission system model, an experimental device for driving the robotic arm joint of the double tendon-sheath transmission system was set up, as shown in Figure 2.

The experimental platform the following: A DC servo motor; 4 pull force sensors (DYMH-106), 2 pull force sensors arranged on each tendon-sheath to collect the pull force at the input and output ends; 2 encoders, respectively, collecting the driving wheel and the angle of the driven wheel; a mechanical arm joint made of aluminium alloy material; and a weight for simulating the end-load of the mechanical arm joint. The flexible cable used in the experiment was a stainless steel wire rope with a 0.8 mm diameter, and an elastic modulus of 1.45 × 10^5^ M pa. The inner diameter of the flexible sleeve was 2 mm, and the inner side was a plastic contact surface. Additionally, the pretension force can be changed by a pretension device. The equipment used is shown in Table 1.

**Figure 3:**
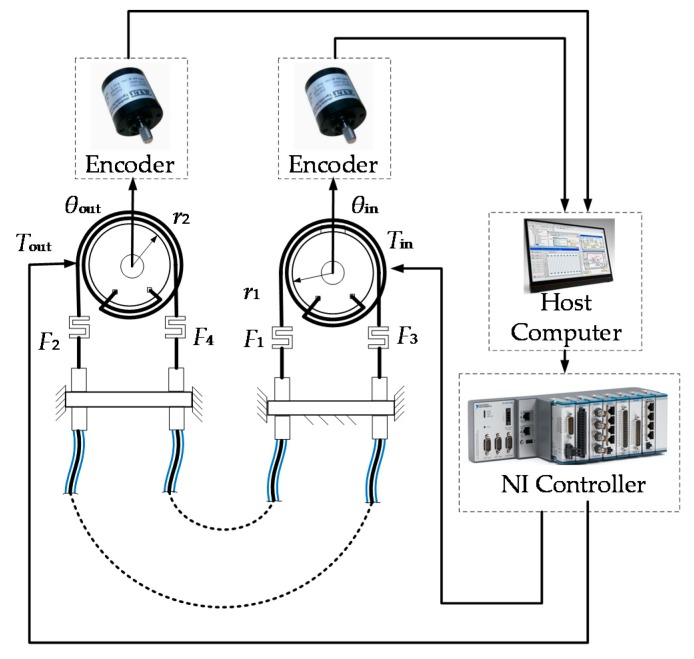
Overall block diagram of the control system.

The experimental schematic diagram of the double tendon-sheath transmission system is shown in Figure 3. The semi-physical real-time control of the system was based on a real-time simulation environment in Labview.

In the development environment of Labview, the rotation angles of the driving wheel and the driven wheel are monitored in real-time. The upper computer was a laptop computer, which communicates with the lower computers using Ethernet. The angle information of the remote and near pulleys was obtained in real-time by the encoder. The control system used NI-cRIO-9067 as the slave computer. The rotational displacement of the motor and joint was measured by an encoder and input to a digital input / output module. The force of the tendon on the joint side was measured by a tension sensor (DYMH-10Kg) and input by the signal amplifier into the analog input module.

### 3.2. Analysis of the Torque Transmission Model of a Double Tendon-Sheath Transmission System

In a double tendon-sheath transmission system, the bending angles of the two tendon-sheath were set to πrad, the diameters of the proximal pulley and the distal pulley were both 30 mm, and the system pre-tightening force was set to approximately 30 N. The position signal at the near end was measured by the encoder built into the motor, and the position output signal at the far end was measured by the encoder on the rotation axis of the single articulated arm.

According to the experimental and simulation results, it can be seen that the curves of the theoretical output torque *T*_out_ and the experimental output torque *T*_exp_ are essentially the same, which proved the correctness of the derived model.

**Figure 4:**
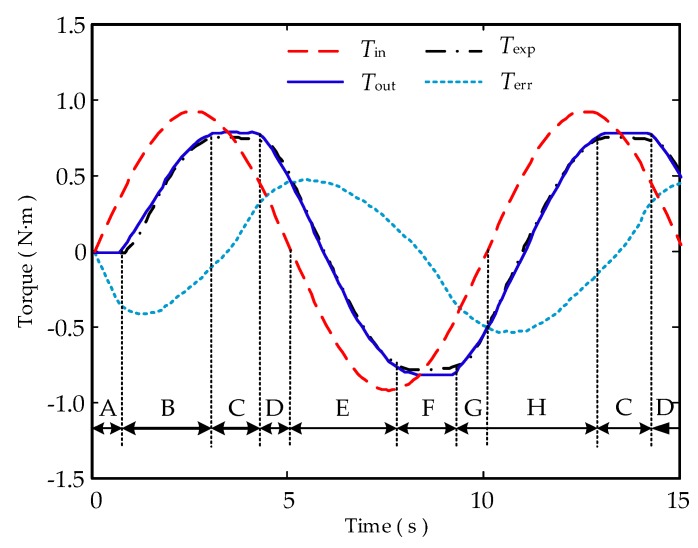
Model analysis of the double tendon-sheath torque transfer characteristics.

To analyse the characteristics of the hysteresis, the air return, and other characteristics of the double tendon-sheath transmission system in detail, according to Equation (17), the torque transmission process of the double tendon-sheath system can be divided into phases A to G, with the clockwise direction being positive and the counterclockwise direction being negative, as shown in Figure 4.

Segment A: When *T*_in_ increases clockwise, *T*_in_*r*_2_/*r*_1_ − *T*_out_ ≤ 2*F*_0_*r*_2_*μΚ*, *T*_out_ = 0, and the system is at a standstill.

Segment B: When *T*_in_ increases clockwise, *T*_in_*r*_2_/*r*_1_ − *T*_out_ > 2*F*_0_*r*_2_*μΚ*, *T*_out_ = *T*_in_*r*_2_/*r*_1_ − 2*F*_0_*r*_2_*μΚ*, and the system rotates clockwise.

Segment C: When *T*_in_ decreases counterclockwise, *T*_out_ − *T*_in_*r*_2_/*r*_1_ ≤ 2*F*_0_*r*_2_*μΚ*, *T*_out_ remains unchanged, and the system is at a standstill.

Segment D: When *T*_in_ decreases counterclockwise, *T*_out_ − *T*_in_*r*_2_/*r*_1_ > 2*F*_0_*r*_2_*μΚ*, *T*_out_ = *T*_in_*r*_2_/*r*_1_ + 2*F*_0_*r*_2_*μΚ*, and the system rotates counterclockwise.

Segment E: When *T*_in_ increases counterclockwise, *T*_out_ − *T*_in_*r*_2_/*r*_1_ > 2*F*_0_*r*_2_*μΚ*, *T*_out_ = *T*_in_*r*_2_/*r*_1_ + 2*F*_0_*r*_2_*μΚ*, and the system rotates counterclockwise.

Segment F: When *T*_in_ decreases clockwise, *T*_in_*r*_2_/*r*_1_ − *T*_out_ ≤ 2*F*_0_*r*_2_*μΚ*, *T*_out_ remains unchanged, and the system is at a standstill.

Segment G: When *T*_in_ increases clockwise, *T*_in_*r*_2_/*r*_1_ − *T*_out_ > 2*F*_0_*r*_2_*μΚ*, *T*_out_ = *T*_in_*r*_2_/*r*_1_ − 2*F*_0_*r*_2_*μΚ*, and the system rotates clockwise.

Segment H: When *T*_in_ increases clockwise, *T*_in_*r*_2_/*r*_1_ − *T*_out_ > 2*F*_0_*r*_2_*μΚ*, *T*_out_ = *T*_in_*r*_2_/*r*_1_ − 2*F*_0_*r*_2_*μΚ*, and the system rotates clockwise.

Two tension sensors were, respectively, arranged on the two tendon-sheaths, and the input tension and the output tension were, respectively, measured by the tension sensors. Data acquisition is performed on the position signal and the tension signal through the data acquisition module. To verify the derived model, a sinusoidal signal with a constant amplitude and a constant frequency is used as the input. Among them, *T*_in_, *T*_out_, *T*_exp_, and *T*_err_ were, respectively, the input driving torque, the theoretical output torque, the experimental output torque, and the difference between the input and output torques.

## 4. Experimental Research on Double Tendon-sheath Transmission System

The requirements of the equipment are different under different working conditions, such as multi-joint manipulators, as the parameters of each joint part may be different, thus the analysis of the transmission characteristics of the tendon-sheath drive system is helpful to the development of the robot. According to Equation (17), the factors affecting the transmission characteristics are the radius of the near pulley *r*_1_, the radius end of the far pulley *r*_2_, the preload force *F*_0_, the friction factor of the noose transmission system *μ*, and the noose bending angle *Κ*. The characteristics of torque transmission and displacement transmission are given under different conditions. 

### 4.1. Analysis of the Transmission Characteristics of the Double Tendon-sheath

(1) Change in the lubricating condition

In this group of experiments, the bending angle of the double tendon-sheath is π, and the motor has a sinusoidal signal with a frequency of 0.1 Hz and a certain amplitude.

**Figure 5:**
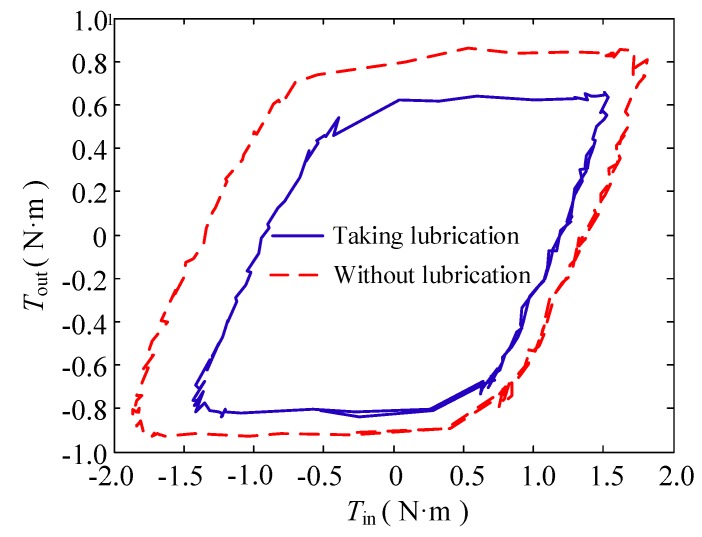
Torque transfer relationship when the lubrication condition changes.

The torque transmission characteristics are shown in Figure 5. Under the condition that the output torque of the double tendon-sheath transmission system is constant, the input torque after lubrication treatment is significantly smaller than that without lubrication, and the transmission gap becomes smaller.

**Figure 6:**
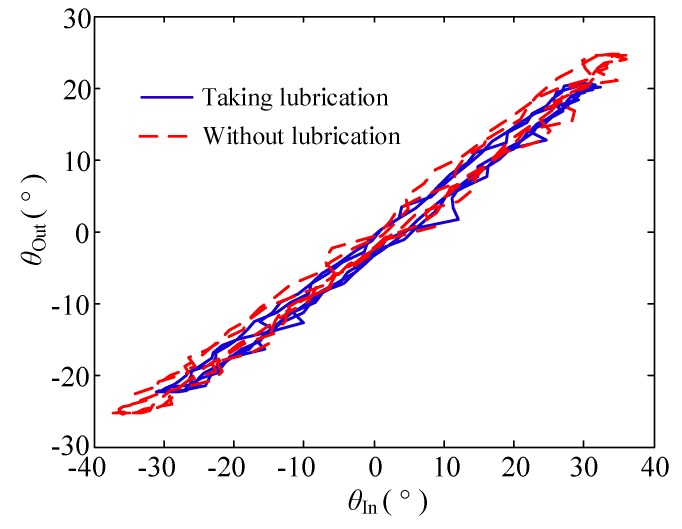
Displacement transfer relationship when the lubrication condition changes.

The displacement transmission characteristics are shown in Figure 6. The displacement transmission gap after lubrication is significantly smaller than that without lubrication.

(2) Change in the pulley radius

In this group of experiments, the bending angle of the double tendon-sheath was set at π, and the motor had a sinusoidal signal with a frequency of 0.1 Hz and a certain amplitude to ensure the same output torque. Two specifications of *r*_1_ = *r*_2_ = 15 mm and *r*_1_ = *r*_2_ = 20 mm are used, and pulleys were used to perform transmission characteristic experiments.

**Figure 7:**
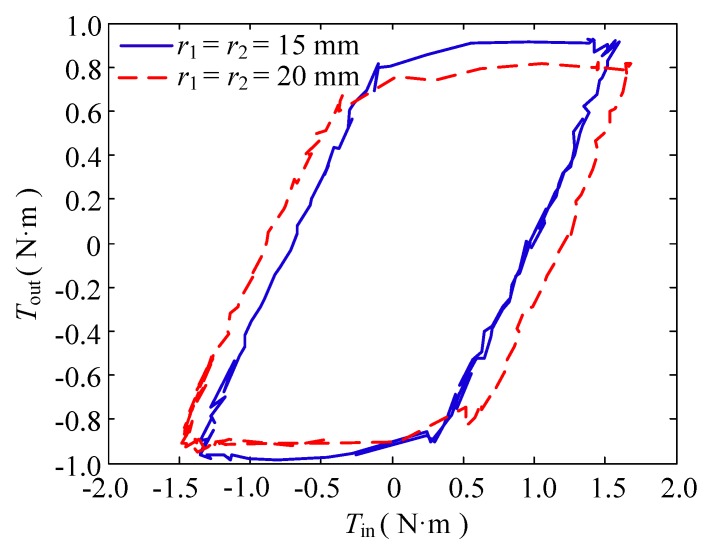
Torque transfer relationship when the radius of the pulley changes.

The results of the torque transmission characteristics are shown in Figure 7. Under the condition that the output torque of the double tendon-sheath transmission system was constant, the clearance of the torque transmission characteristics was obviously smaller than that when *r*_1_ = *r*_2_ = 20 mm.

**Figure 8:**
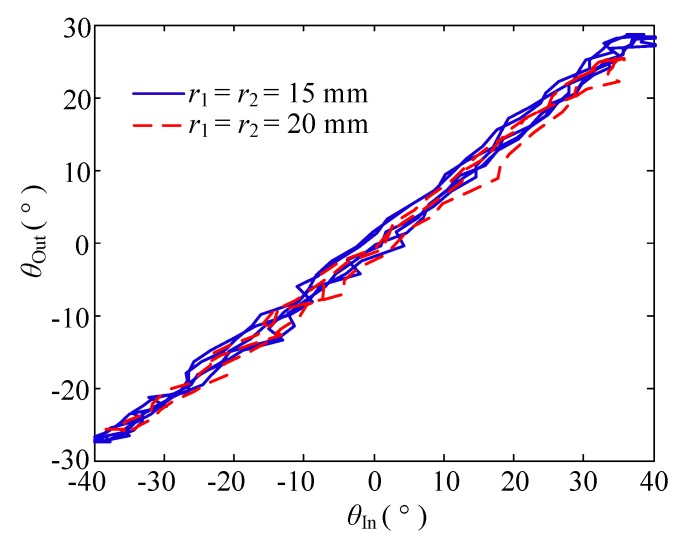
Displacement relationship when the radius of the pulley changes.

The displacement transmission characteristics are shown in Figure 8. The displacement transmission gap when *r*_1_ = *r*_2_ = 15 mm was significantly smaller than when *r*_1_ = *r*_2_ = 20 mm.

### 4.2. Analysis of the Torque Transmission Efficiency of the Double Tendon-Sheath Transmission System

To improve the torque transmission efficiency of the double tendon-sheath transmission system, it can be seen from the analysis of the torque transmission characteristics and the displacement transmission characteristics that the transmission efficiency can be improved by reducing the friction torque between the casing and the flexible cable, that is, by reducing the size of 2*F*_0_*r*_2_*μΚ*. On the experimental platform of the double tendon-sheath driven single joint manipulator, the parameters of the experimental platform pretension force, the full tendon-sheath curvature, the input and output pulley radii, and the friction factor were adjusted, respectively. The system is determined by *θ*_in_ = π/4sin(π/5*t*) sine, and the trajectory drives the robotic arm to swing.

#### 4.2.1. Decrease the Preload of the System

**Figure 9:**
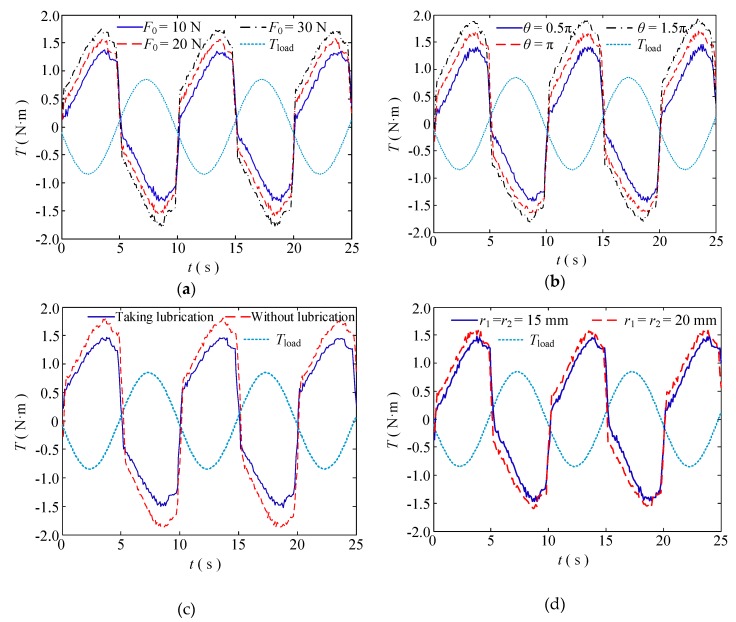
Transmission efficiency analysis for the double tendon-sheath torque; (**a**) torque transmission efficiency with different preload; (**b**) moment transfer efficiency at full curvature of different tendon-sheathes; (**c**) torque transmission efficiency under different friction factors; (**d**) torque transmission efficiency with different preload.

In the experiment, the noose was fixed thus that the total curvature *Κ* was π rad. There was no lubrication between the casing and the flexible noose, and the pulley radius *r*_1_ = *r*_2_ = 15 mm. The preload force of the system is adjusted to 10 N, 20 N, and 30 N. The experimental results are shown in Figure 9a. Under the same load conditions, the smaller the preload force, the smaller the normal pressure between the sleeve and the flexible cable, and the smaller the friction torque during the transmission process. However, if the preload force is too small, the tendon-sheath will loosen during the transmission process. Therefore, the system’s pretension needs to be reasonably set. For the double tendon-sheath transmission system under different configuration conditions, the minimum pretension should be analysed.

#### 4.2.2. Reduce the System’s Full Curvature

The pre-tightening force of the double tendon-sheath transmission system was set to 25 N. There was no lubrication between the sleeve and the flexible cable, and the pulley radius *r*_1_ = *r*_2_ = 15 mm. The total curvature of the casing is adjusted to 0.5π, π, and 1.5π, respectively, with three sets of experiments performed, and the influence of the variation of the casing’s full curvature on the transmission efficiency analysed. The experimental results are shown in Figure 9b. 

When the load was constant, the smaller the total curvature of the casing, that is, the smaller the bending angle, the smaller the driving torque required, and the higher the transmission efficiency. Therefore, in the application of actually driving the robotic arm, the bending angle of the transmission tendon-sheath can be reduced by reasonably arranging the positions of the driving motor and the load end, thereby improving the transmission efficiency and reducing the requirements of the driving device.

#### 4.2.3. Reduce the Coefficient of Friction

The tendon sheath-driven transmission system has a full curvature set to πrad, the system’s pretension force is adjusted to 30 N, and the pulley radius *r*_1_ = *r*_2_ = 15 mm. In the first set of experiments, there is dry friction between the flexible cable and the casing, and in the second set of experiments, grease was applied between the casing and the flexible cable in order to reduce the friction coefficient of the transmission system. The experimental results are shown in Figure 9c. Under the condition of a certain load, the driving torque required by the tendon sheath-driven transmission mechanism after lubrication was less than that without lubrication. Therefore, in a practical applications, the transmission efficiency can be improved by taking reasonable lubrication measures.

#### 4.2.4. Reduce the Pulley Radius

In the experiment, the full curvature of the tendon-sheath was set to πrad. the system pretension force was 25 N, and there was dry friction between the flexible cable and the casing. Two pulleys, *r*_1_ = *r*_2_ = 15 mm and *r*_1_ = *r*_2_ = 20 mm were used to carry out the transfer efficiency experiment. As shown in Figure 9d, under a certain load condition, the smaller the radius of the pulley, the greater the pull-up force of the flexible cable, and the smaller the required driving torque, but with higher requirements on the drive system. Therefore, in practical applications, the radius of the pulley can be reasonably configured according to the load to improve the system efficiency.

## 5. Position Compensation Control of the Double Tendon-Sheath Drive System

The offline compensation method is suitable for cases where it is inconvenient to set a sensor at the end. Although no feedback at the output is required, it is usually performed under a certain configuration environment, thus it is more limited in practical applications. During the actual movement of the robotic arm, the bending angle of the tendon-sheath will change with the movement of the robotic arm. Thus the offline compensation control method is not suitable. To achieve a good control effect, a suitable method for transmission of the compensation control scheme in the case of sudden changes in characteristics requires a force or displacement sensor at the end of the signal feedback. Considering the motion form of the robot arm joint, an encoder can be installed on the rotary axis of the robot arm joint to collect the end position signal in real-time to achieve position feedback. Therefore, a small encoder is installed on the robot arm joint test bench on the double tendon-sheath driving robot arm joint to perform real-time signal feedback compensation control experiments.

### 5.1. Compensation Control Model

From the torque transmission model and displacement transmission model, it can be determined that during the movement of the tendon-sheath transmission system, friction between the casing and the flexible cable as well as elastic deformation of the flexible cable tension will cause a phase lag and a transmission gap. The existence of the torque transmission gap and the displacement transmission gap will lead to a reduction in accuracy, especially in the process of changing the transmission direction. Although the gap can be reduced by adjusting the pretension force, etc., it cannot be eliminated, thus it needs to be compensated and controlled.

**Figure 10:**
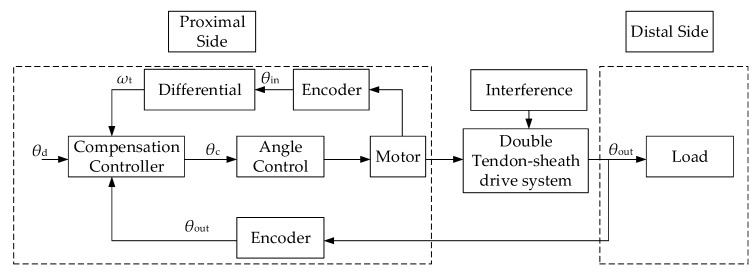
Compensation control model of a double tendon sheath transmission system.

During the actual movement of the robotic arm, the position and bending angle of the double tendon-sheath transmission system corresponding to the joint are changing. Based on the analysis of the previous transmission characteristics, it can be determined that when the external environment changes, such as the tendon-sheath, as the bending angle of the beam changes, and its transmission characteristics also change. Therefore, a double tendon-sheath transmission system should be successfully applied to the robotic arm, and the torque transmission efficiency and the transmission accuracy should be improved to the greatest extent possible to reduce the torque tracking error. The position compensation control scheme based on the end signals feedback design is shown in Figure 10. In the position control compensation scheme, the actual input position signal of the system is the sum of the required output position and the compensation error.

The compensation controller is set to

(25) $$\theta_{c}=\theta_{d}+\theta_{f}$$

where *θ*_d_, *θ*_f,_ and *θ*_c_, represent the desired tracking trajectory of the output angle, the amount of friction compensation, and the actual input instructions that the angle controller needs to follow, respectively,

(26) $$\theta_{f}=\left\{ \begin{array}{l} \frac{\varphi_{s}\left(t\right)r_{2}}{r_{1}},\omega_{\mathrm{in}}\left(t\right)>0\\ \frac{\varphi_{n}\left(t\right)r_{2}}{r_{1}},\omega_{\mathrm{in}}\left(t\right)<0\\ \theta_{f}\left(t^{-1}\right),\omega_{\mathrm{in}}\left(t\right)=0 \end{array}\right.$$

### 5.2. Compensation Control Experiments

To verify the effectiveness of the proposed position compensation controller, a torque tracking experiment was performed. To simulate the change of the position and bending angle in the manipulator during the movement of the external arm when the configuration environment is changed, the configuration of the double tendon-sheath transmission system is changed manually and randomly during the experiment. The experimental operator manually moved the tendon-sheath during the experiment to make the transmission line fluctuate. The tendon-sheath then moved randomly in three directions: Up, down, back, and left to change its route to the greatest extent possible (within the limit that does not cause any tendon-sheath to fold). In the course of the lanyard route fluctuation, the condition of the contact between the flexible cable and the casing changes to the greatest extent possible to simulate the working situation of the robot arm during the actual movement.

**Figure 11:**
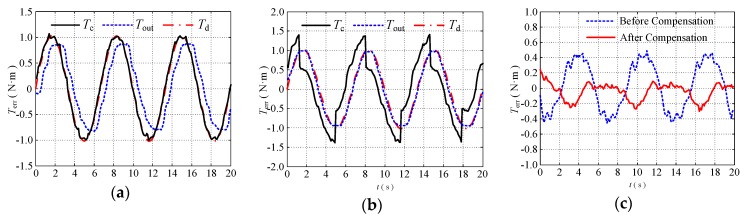
Torque tracking experiment with 2.5 kg end load under external disturbance; (**a**) before compensation; (**b**) after compensation; (**c**) torque tracking error.

**Figure 12:**
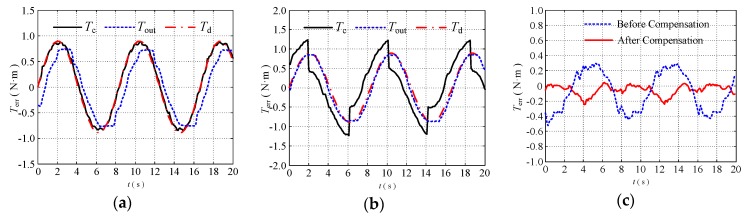
Torque tracking experiment with 2 kg end load under external disturbance; (**a**) before compensation; (**b**) after compensation; (**c**) torque tracking error.

The proposed compensation controller is a closed-loop compensation controller. When the load is changed, its controller parameters need to be changed to ensure the performance of the control strategy. To verify that the system can achieve a good control performance under different loads, three sets of experiments were performed to change the end-load of the single-joint robotic arm, and the weights of 2.5 kg and 2 kg weights were replaced at the end of the single-joint robotic arm, respectively. Different controller parameters for torque tracking experiments were set under different loads. The experimental results are shown in Figure 11 and Figure 12.

The experimental tracking error is shown in Figure 11c and Figure 12c.

(1) With a load of 2.5 kg, the average tracking error of the torque decreased from 43.51% (without compensation) to 13.30% (with compensation);

(2) When the load was 2 kg, the average tracking error of the torque was reduced from 41.30% (without compensation) to 10.67% (with compensation);

Therefore, the position control compensator has a good compensation effect, and the control method can be used in a humanoid manipulator driven by a double tendon-sheath.

## 6. Conclusions

During the working process of the double tendon-sheath transmission system, there is a gap between its torque transmission and its displacement transmission. According to the transmission model and experimental analyses, it can be determined that reducing the preload force *F*_0_, can reduce the output pulley radius *r*_2_, and decrease the sleeve. The friction coefficient of the cable *μ* and measures that reduce the total curvature *Κ* of the cable are used to improve the torque transmission efficiency of the dual cable system. In addition, for a tendon sheath-driven transmission system to operate normally, the pretension force in its initial stage must be higher than the minimum pretension force.

When the manipulator is working, the configuration of the double tendon-sheath transmission system can change at any time. Therefore, a double tendon-sheath driven robot arm joint is designed based on the double tendon-sheath transmission mechanism and the joint motion form of the robot arm. A compensation control method with a good compensation effect based on encoder position feedback was proposed. This method can be used in a humanoid manipulator driven by a double tendon-sheath. After a lot of research, we successfully developed the first generation of tendon-sheath-driven dexterous hands and robotic arm. Please refer to Video S1.

## Figures and Tables

**Table 1:** The main hardware of the double tendon-sheath transmission experiment system.

Device	Specification	Quantity	Supplier
Rouzo	0.8 mm diameter	2	DEKDEJA
Casing	2 mm in diameter	2	TRLREQ
Drive pulley	30 mm diameter	2	Custom parts
DC	Output torque 38 N·m	1	ZHUOYUEMOXING
Controller chassis	cRIO-9067	1	Custom parts
Host computer	computer	1	DELL
Encoder	12-bit absolute encoder	2	YOGOMO
Tension sensor	DYMH-106	4	DYMH
Voltage module	NI-9205, NI-9264	1	NI

## Supplementary Materials

The following are available online at https://www.mdpi.com/1424-8220/20/5/1301/s1, Video S1: Tendon-sheath driven dexterous hand and humanoid robotic arm.
